# Incidence and outcomes of in-hospital cardiac arrest in Japan 2011–2017: a nationwide inpatient database study

**DOI:** 10.1186/s40560-022-00601-y

**Published:** 2022-03-03

**Authors:** Hiroyuki Ohbe, Takashi Tagami, Kazuaki Uda, Hiroki Matsui, Hideo Yasunaga

**Affiliations:** 1 Department of Clinical Epidemiology and Health Economics, School of Public Health, The University of Tokyo, 7-3-1 Hongo, Bunkyo-ku, Tokyo, 1130033 Japan; 2 Department of Emergency and Critical Care Medicine, Nippon Medical School, Musashi-Kosugi Hospital, 1-396 Kosugimachi, Nakahara-ku, Kawasaki-shi, Kanagawa 2118533 Japan; 3 University of Tsukuba, 1-1-1 Tennoudai, Tsukuba, Ibaraki 3058575 Japan

**Keywords:** In-hospital cardiac arrest, Epidemiology, Incidence, Cardiac arrest, Administrative database

## Abstract

**Background:**

Although numerous studies have investigated out-of-hospital cardiac arrest, few studies have been conducted on in-hospital cardiac arrest (IHCA). Knowledge of the nationwide epidemiology of IHCA in Japan, with its super-aging society, is important to understand the current situation of IHCA and to establish evidenced-based medicine in the future. The present study aimed to determine the incidence and outcomes of IHCA and their trends in Japan.

**Methods:**

This observational cohort study was performed using a national administrative inpatient database for more than 1600 acute-care hospitals covering about 50% of all acute-care hospital beds in Japan from April 2011 to March 2018. We defined cardiac arrest patients who received cardiopulmonary resuscitation (chest compression) during hospitalization as IHCA. We excluded out-of-hospital cardiac arrest patients from the source population. The incidence of IHCA per 1000 hospital admissions and survival to discharge rate was reported with trend analyses by calendar year 2011–2017.

**Results:**

Among 53,871,101 hospitalized patients without out-of-hospital cardiac arrest patients in 1626 hospitals, 2,136,038 (4.0%) had cardiac arrest. Of them, 274,664 (12.9%) received cardiopulmonary resuscitation at least once during hospitalization and were identified as IHCA, and 1,861,374 (87.1%) did not receive cardiopulmonary resuscitation. The incidence of IHCA per 1000 hospital admissions was 5.1, with a significant decreasing trend from 6.1 in 2011 to 4.6 in 2017 (*P* for trend = 0.033). Our estimated incidence can be translated to approximately 87,000 IHCA cases in Japan each year. The percentage of IHCA patients among cardiac arrest patients was 12.9%, with a significant decreasing trend from 14.0% in 2011 to 12.2% in 2017 (*P* for trend = 0.006). The overall rate of survival to discharge was 12.7%, with a significant increasing trend from 10.5% in 2011 to 14.0% in 2017 (*P* for trend < 0.001).

**Conclusions:**

We found substantial associations between mortality and loss of health and IHCA in Japan. The incidence of IHCA showed a decreasing trend over time, the percentage of treated cardiac arrest patients also had a decreasing trend, and the overall survival to discharge rate improved over time.

**Supplementary Information:**

The online version contains supplementary material available at 10.1186/s40560-022-00601-y.

## Background

In-hospital cardiac arrest (IHCA) is an acute event that can occur in any patient during hospitalization [1]. IHCA was reported to be associated with high morbidity and mortality [1–3].

Although numerous studies have investigated out-of-hospital cardiac arrest (OHCA) [4–6], few studies have been conducted on IHCA. To date, there are only two nationwide prospective registries for IHCA: the American Heart Association Get with the Guidelines-Resuscitation registry in the United States [7] and the Resuscitation Council and Intensive Care National Audit and Research Centre in the United Kingdom [8]. The majority of the evidence on IHCA is derived from these two registries [1], and the global incidence of IHCA has not been well described [9, 10]. In Japan, only two multi-centre studies involving 491 and 228 IHCA patients have been performed [11, 12], and there is currently no nationwide prospective registry for IHCA.

Knowledge of the nationwide epidemiology of IHCA in Japan, with its super-aging society, is important to understand the current situation of IHCA and to establish evidenced-based medicine in the future [9]. Therefore, the present study aimed to determine the incidence and outcomes of IHCA and their trends in Japan using a national administrative inpatient database.

## Methods

### Study design and data

This was a nationwide retrospective cohort study using a national administrative inpatient database in Japan. Specifically, we used the Japanese Diagnosis Procedure Combination database, which contains discharge abstract and administrative claims data from more than 1600 acute-care hospitals that voluntarily contribute to the database and cover approximately 50% of all acute-care hospital beds in Japan [13]. The database contains the following patient-level data for all hospitalizations: age; sex; dates of hospitalization and discharge; diagnoses recorded with International Classification of Diseases, Tenth Revision (ICD-10) codes and text in Japanese; surgical and nonsurgical procedures and dates of the procedures; dates and doses of drugs administered during hospitalization; and discharge status. In a previous study on the validity of the diagnoses and procedures recorded in the database, the specificity of recorded diagnoses exceeded 90%, the sensitivity of recorded diagnoses ranged from 50 to 80%, and the specificity and sensitivity of recorded procedures exceeded 90% [14].

The present study was approved by the Institutional Review Board of The University of Tokyo (Approval number 3501-3; 25 December 2017). Given the de-identified nature of the data, the requirement for informed consent was waived.

### Study population

Using the Japanese Diagnosis Procedure Combination database, we identified all hospitalized patients from April 2011 to March 2018. Based on the consensus definition for IHCA in 2018 [15, 16], patients with OHCA, defined as ICD-10 codes I46.x (cardiac arrest) in the admission diagnosis, were excluded from the study population. Of the remaining patients, non-cardiac arrests patients were excluded and cardiac arrest patients, defined as those who received cardiopulmonary resuscitation or died during hospitalization, were included. For this study, IHCA patients were defined as those who had undergone cardiopulmonary resuscitation (chest compression) at least once during hospitalization [15, 16]. Cardiopulmonary resuscitation was identified by the Japanese procedure code J046 (closed chest compression). Non-treated cardiac arrest was defined as death in hospital without cardiopulmonary resuscitation.

### Incidence

The incidence of IHCA per 1000 hospital admissions was calculated as the number of IHCA patients divided by the number of hospitalized patients among the study population, multiplied by 1000 [15, 16]. The incidence of hospital deaths per 1000 hospital admissions was calculated as the number of hospital deaths divided by the number of hospitalized patients among the study population, multiplied by 1000.

### Outcomes

The primary outcome was survival to discharge. The secondary outcome was favourable neurological outcome at discharge, defined as alert consciousness on the Japan Coma Scale at discharge [17].

### Statistical analysis

To evaluate trends by calendar year, we performed the Cochran–Armitage test for binary variables and the Jonckheere–Terpstra test for continuous variables [18, 19]. The primary outcome was reported for the overall cohort and separately according to defibrillation or extracorporeal cardiopulmonary resuscitation (ECPR) [15], age category, and illness category.

Data were complete for all variables. All analyses were performed using STATA/MP software version 16.0 (STATA Corp, College Station, TX, USA). All hypothesis tests were two-sided, with a significance level of 0.05.

## Results

**Fig. 1:**
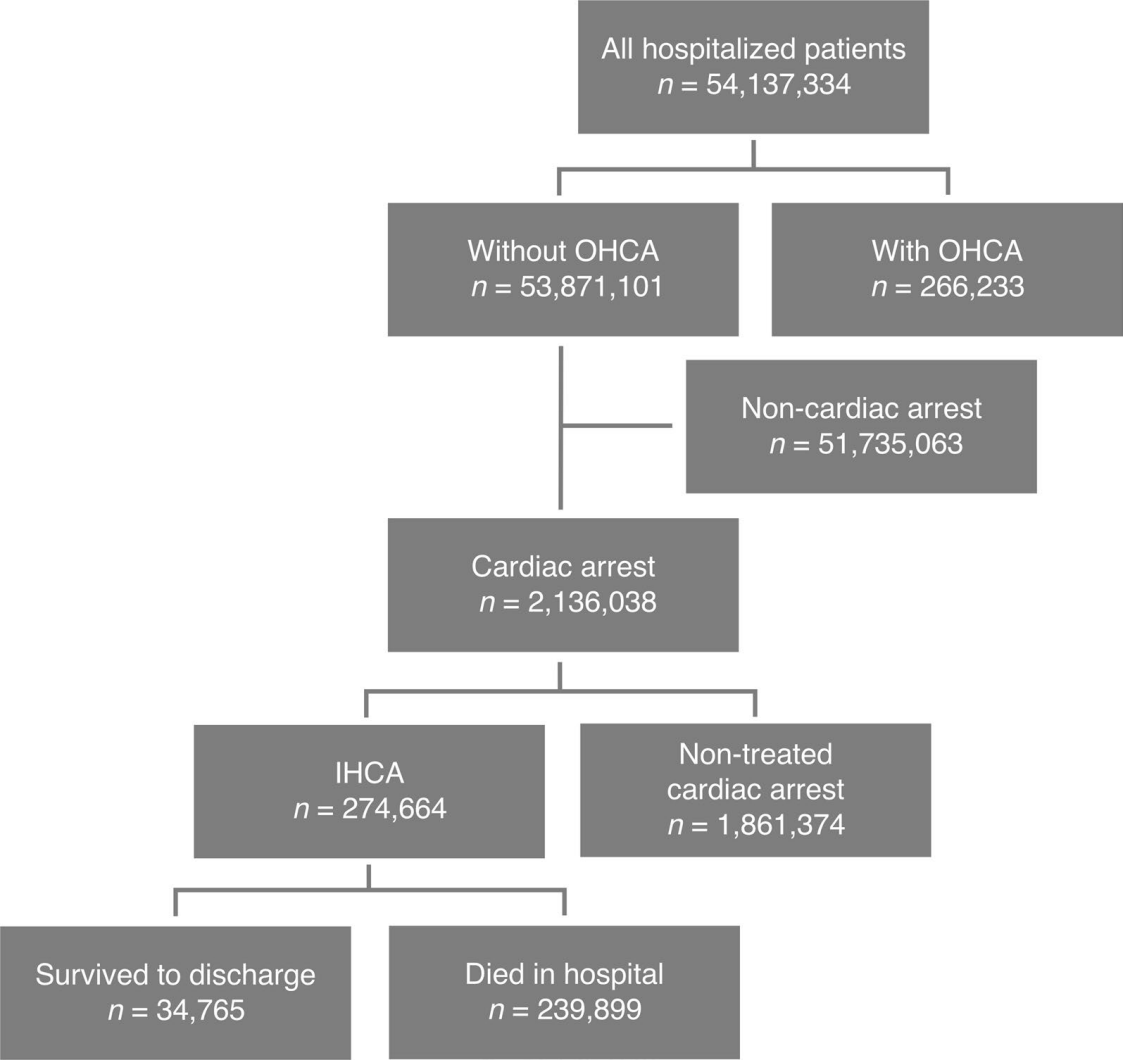
Patient flowchart. *OHCA* out-of-hospital cardiac arrest, *IHCA* in-hospital cardiac arrest

Among 53,871,101 hospitalized patients without OHCA from 1626 hospitals, 2,136,038 (4.0%) had cardiac arrests, defined as patients who received cardiopulmonary resuscitation or died during hospitalization (Fig. 1). Of these, 274,664 (12.9%) received cardiopulmonary resuscitation at least once during hospitalization and were identified as IHCA, and 1,861,374 (87.1%) did not receive cardiopulmonary resuscitation.

The incidence of IHCA per 1000 hospital admissions was 5.1, with a significant decreasing trend from 6.1 in 2011 to 4.6 in 2017 (*P* for trend = 0.033) (Table 1). The incidence of hospital deaths per 1000 hospital admissions was 39.0, with a significant decreasing trend from 43.0 in 2011 to 37.5 in 2017 (*P* for trend = 0.004). The percentage of IHCA patients among cardiac arrest patients was 12.9%, with a significant decreasing trend from 14.0% in 2011 to 12.2% in 2017 (*P* for trend = 0.006).

**Table 1:** Incidence and trends for in-hospital cardiac arrest

	Total	Fiscal year							*P* for trend
		2011	2012	2013	2014	2015	2016	2017	
Number of hospitals, *n*	1626	1067	1072	1061	1189	1262	1332	1253	–
Hospitalized patients without OHCA, *n*	53,871,101	6,746,300	7,116,012	7,076,246	8,030,725	8,176,964	8,500,726	8,224,128	–
Hospital deaths among study population, *n*	2,101,273	289,770	282,379	270,582	311,287	313,108	325,461	308,686	–
Non-treated cardiac arrests, *n*	1,861,374	252,842	247,420	239,193	276,492	279,546	290,091	275,790	–
IHCA, *n*	274,664	41,263	39,810	35,927	39,866	38,734	40,827	38,237	–
Incidence of hospital deaths per 1000 admissions	39.0	43.0	39.7	38.2	38.8	38.3	38.3	37.5	0.033
Incidence of IHCA per 1000 admissions	5.1	6.1	5.6	5.1	5.0	4.7	4.8	4.6	0.004
Percentage of IHCA among cardiac arrest patients, %	12.9	14.0	13.9	13.1	12.6	12.2	12.3	12.2	0.006

Each fiscal year started on 01 April and ended on 31 March

*OHCA* out-of-hospital cardiac arrest, *IHCA* in-hospital cardiac arrest

The patient characteristics and their trends for IHCA are shown in Table 2. The mean age was 73.2 ± 17.1 years and 60.5% were male. More than half the patients were over 75 years old. The most common illness category was medical—noncardiac (55.0%), followed by medical—cardiac (29.4%). In the cardiac arrest process, defibrillation and ECPR were delivered in 14.4% and 3.4% of patients, respectively. In the post-resuscitation phase, 24.9% and 12.6% of patients required intensive and high dependency care unit admission, respectively. Targeted temperature management and percutaneous coronary intervention were performed in 1.3% and 4.1% of patients, respectively. The IHCA population gradually became older. There was a marked increase in the percentage of patients over 85 years old. The proportions of patients with medical—cardiac, surgical—cardiac, and trauma increased over time, while the proportions of patients with medical—noncardiac and surgical—noncardiac decreased. Regarding the cardiac arrest process, the proportion of patients who received defibrillation decreased, while the proportion of patients who received ECPR increased. All investigated post-resuscitation processes, including intensive and high dependency care unit admission, increased over time.

**Table 2:** Patient characteristics and their trends for in-hospital cardiac arrest

	Total	Fiscal year							*P* for trend
		2011	2012	2013	2014	2015	2016	2017	
Demographic characteristics									
Age (years), mean	73.2	73.3	72.7	72.4	73.1	73.2	73.6	73.9	< 0.001
Infants (aged < 1 year), %	1.1	1.0	1.2	1.4	1.2	1.1	1.1	0.9	0.065
Children (aged 1–18 years), %	1.0	0.8	1.0	1.0	1.0	1.0	1.0	0.9	0.734
Adults									
Aged 19–64 years, %	19.1	20.1	20.4	20.1	18.9	18.5	18.0	17.8	< 0.001
Aged 65–74 years, %	21.2	20.1	20.8	21.4	21.5	22.1	21.2	21.4	< 0.001
Aged 75–84 years, %	32.8	34.2	33.6	33.0	32.5	32.4	32.2	31.7	< 0.001
Aged > 84 years, %	24.8	23.8	23.0	23.0	24.9	24.7	26.5	27.2	< 0.001
Male sex, %	60.5	59.9	60.6	61.0	60.5	60.6	60.4	60.4	0.571
Illness category, %									
Medical—cardiac	29.4	27.0	27.6	28.6	29.2	29.8	31.0	32.4	< 0.001
Medical—noncardiac	55.0	58.7	56.4	55.3	55.1	54.2	53.3	51.8	< 0.001
Surgical—cardiac	2.8	2.4	2.9	2.9	2.8	3.1	2.8	3.1	< 0.001
Surgical—noncardiac	6.3	6.3	6.7	6.6	6.2	6.2	5.9	6.0	< 0.001
Obstetric	0.1	0.0	0.1	0.1	0.1	0.1	0.1	0.0	0.974
Trauma	6.5	5.7	6.4	6.5	6.7	6.6	6.9	6.7	< 0.001
Cardiac arrest process									
Arrest on weekend, %	27.4	27.2	27.6	27.3	27.6	27.2	27.5	27.4	0.802
Automated external defibrillator, %	2.3	2.1	2.1	2.0	2.3	2.4	2.5	2.5	< 0.001
Defibrillation, %	14.4	14.8	14.7	14.6	14.5	14.3	13.9	14.1	< 0.001
Extracorporeal cardiopulmonary resuscitation, %	3.4	2.5	2.9	3.3	3.4	3.8	3.7	4.1	< 0.001
Adrenaline, %	69.7	69.6	68.7	69.5	69.4	69.8	70.3	70.4	< 0.001
Dose of adrenaline (mg), mean	4.8	4.8	4.7	4.9	4.9	4.9	4.8	4.8	< 0.001
Endotracheal intubation, %	51.9	52.6	51.8	51.5	51.9	51.7	51.9	52.2	0.597
Post-resuscitation process									
Intensive care unit admission, %	24.9	20.9	22.7	23.8	24.4	25.0	24.1	24.4	< 0.001
High dependency care unit admission, %	12.6	12.2	14.2	15.8	16.2	16.9	19.7	20.7	< 0.001
Targeted temperature management, %	1.3	0.9	1.2	1.5	1.4	1.5	1.4	1.4	< 0.001
Coronary angiography, %	2.0	1.4	1.7	2.0	1.9	2.2	2.3	2.4	< 0.001
Percutaneous coronary intervention, %	4.1	3.5	3.7	4.1	4.2	4.5	4.3	4.6	< 0.001

Each fiscal year started on 01 April and ended on 31 March

**Fig. 2:**
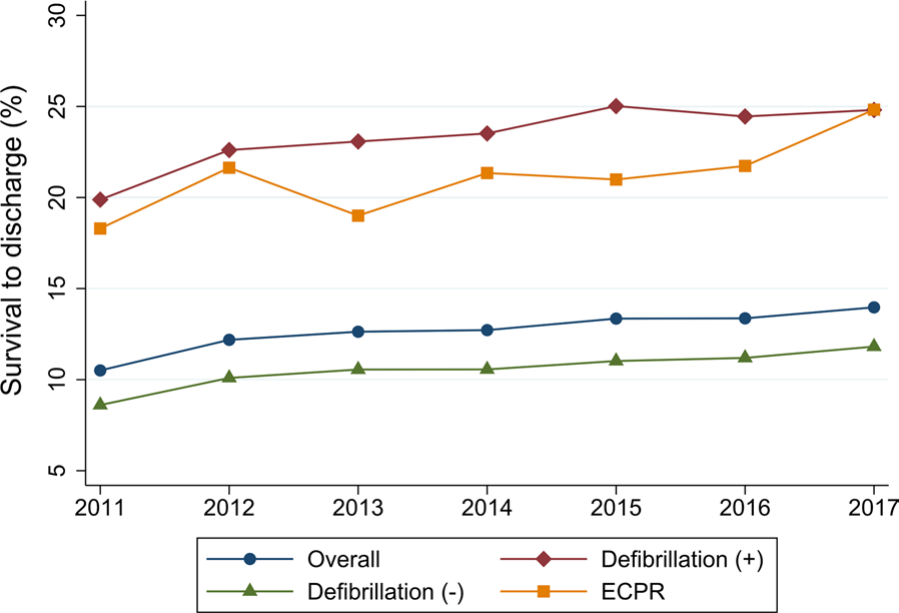
Trends in survival to discharge for the overall cohort and separately according to use of defibrillation or ECPR. Each fiscal year started on 01 April and ended on 31 March. *ECPR* extracorporeal cardiopulmonary resuscitation

**Fig. 3:**
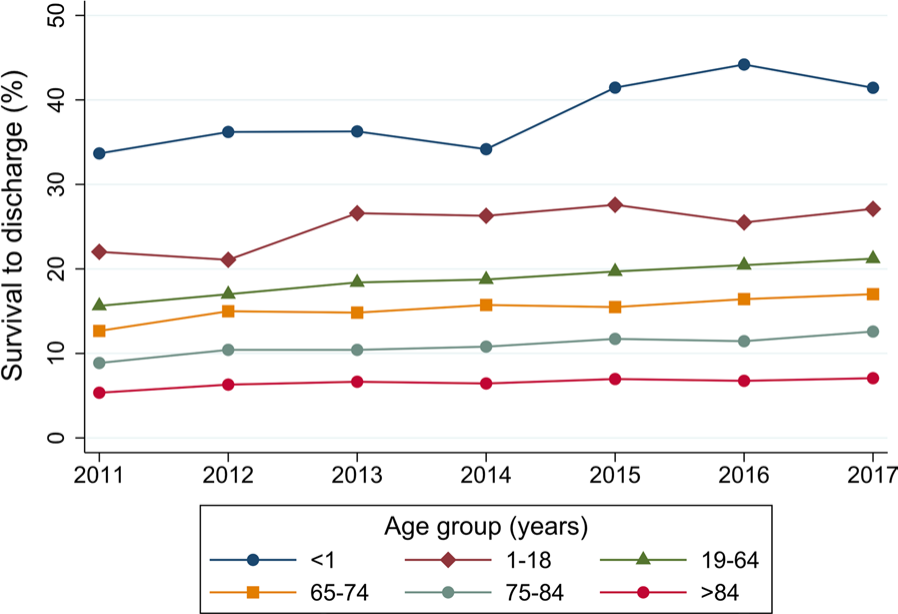
Trends in survival to discharge according to age category. Each fiscal year started on 01 April and ended on 31 March

**Fig. 4:**
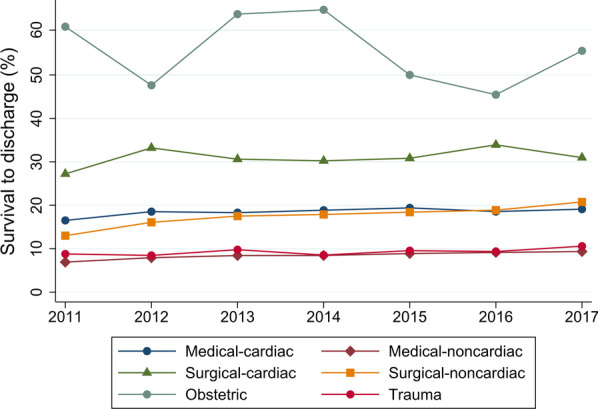
Trends in survival to discharge according to illness category. Each fiscal year started on 01 April and ended on 31 March

The overall rate of survival to discharge was 12.7% (34,765 of 274,664 patients). The rate of survival to discharge increased over time from 10.5% in 2011 to 14.0% in 2017 (*P* for trend < 0.001) (Table 3, Fig. 2, and Additional file 1: Table S1). The rates of survival to discharge among patients with defibrillation and ECPR were 23.3% and 21.3%, respectively. The rates of survival to discharge decreased with increasing age from 38.0% in infants to 6.5% in patients aged > 84 years. The survival rate was lowest in the medical—noncardiac category (8.4%), followed by trauma (9.3%) and surgical—noncardiac (17.4%). There were similar significant trends toward increased survival over time for all subgroups except surgical–cardiac and obstetric (Table 3 and Figs. 2, 3 and 4).

**Table 3:** Survival to discharge and trends for in-hospital cardiac arrest

	Total	Fiscal year							*P* for trend
		2011	2012	2013	2014	2015	2016	2017	
Survival to discharge, %									
Overall	12.7	10.5	12.2	12.6	12.7	13.4	13.4	14.0	< 0.001
Patients with defibrillation	23.3	19.9	22.6	23.1	23.5	25.0	24.5	24.8	< 0.001
Patients without defibrillation	10.5	8.6	10.0	10.6	10.6	11.0	11.2	11.8	< 0.001
Patients with ECPR	21.3	18.3	21.6	19.0	21.3	21.0	21.7	24.8	< 0.001
Age category									
Aged < 1 year	38.0	33.7	36.2	36.3	34.2	41.5	44.2	41.4	< 0.001
Aged 1–18 years	25.2	22.0	21.1	26.6	26.3	27.6	25.5	27.1	0.035
Aged 19–64 years	18.6	15.6	17.0	18.4	18.8	19.7	20.4	21.2	< 0.001
Aged 65–74 years	15.3	12.7	15.0	14.8	15.7	15.5	16.4	17.0	< 0.001
Aged 75–84 years	10.9	8.9	10.4	10.4	10.8	11.7	11.4	12.6	< 0.001
Aged > 84 years	6.5	5.4	6.3	6.6	6.4	7.0	6.8	7.1	< 0.001
Illness category									
Medical—cardiac	18.4	16.5	18.5	18.3	18.9	19.4	18.6	19.1	< 0.001
Medical—noncardiac	8.4	6.9	7.9	8.4	8.4	8.9	9.1	9.4	< 0.001
Surgical—cardiac	31.1	27.2	33.2	30.6	30.2	30.8	33.9	31.0	0.11
Surgical—noncardiac	17.4	13.0	16.1	17.5	17.9	18.4	18.9	20.8	< 0.001
Obstetric	55.6	61.1	47.6	64.0	65.0	50.0	45.5	55.6	0.53
Trauma	9.3	8.8	8.5	9.8	8.5	9.6	9.4	10.6	0.023

Each fiscal year started on 01 April and ended on 31 March

*ECPR* extracorporeal cardiopulmonary resuscitation

## Discussion

To the best of our knowledge, this is the first nationwide epidemiological study of IHCA in Japan. The incidence of IHCA was 5.1 per 1000 hospital admissions from 2011 to 2017. The incidence of IHCA showed a decreasing trend over time, and the percentage of treated cardiac arrest patients also had a decreasing trend. The overall rate of survival to discharge for IHCA was 12.7%, and improved over time.

The incidence of IHCA per 1000 hospital admissions in the present study was 5.1. Given that the reported annual number of hospital admissions in all acute-care hospitals in Japan in 2017 was approximately 17,000,000 [20], our estimated incidence can be translated to approximately 87,000 IHCA patients in Japan each year. The reported annual numbers of total OHCA events and witnessed OHCA events in Japan were 120,000 and 25,000, respectively, in 2017 [21]. Therefore, the present findings indicate that IHCA is an important public health issue like OHCA, but is an area that has received less attention than OHCA. Given that the hospitals included in the database used in this study are acute-care hospitals in which many critically ill patients are treated, the above may be an overestimate and should be treated with caution.

The incidence of IHCA in Japan decreased over time, while the incidence of IHCA in the Unites States increased during the same period [22]. Because the population in Japan is aging and the total number of deaths is increasing [23], our result for the declining incidence of IHCA was unexpected. There are several possible explanations. First, the incidence of hospital deaths per 1000 admissions also decreased, suggesting that the number of people who died outside of acute-care hospitals may have been increasing over time. According to the Vital Statistics in Japan, in-hospital deaths reached 83% in 2005 and then gradually declined to 75% in 2017, while nursing home deaths accounted for 2% in 2005 and 10% in 2017 [24]. Second, the percentage of treated cardiac arrest patients decreased, suggesting that the number of patients with do-not-resuscitate orders may have been increasing over time. Previously in Japan, cardiopulmonary resuscitation and life-sustaining support would often be performed despite their futility [25]. Over the past decade, newly developed guidelines may have changed the practice for end-of-life care in Japan [25]. Third, the introduced rapid-response system may have decreased cardiac arrests in hospital [26, 27].

Although the outcomes remained poor, the present study suggests improvement of the outcomes for IHCA over time. The trends toward improved outcomes for IHCA in our study were similar to those in a previous nationwide study in the United States and a systematic review and meta-analysis [28, 29]. We also found that survival after IHCA improved regardless of whether or not patients received defibrillation. The following factors may have improved the outcomes: earlier detection of cardiac arrest, improved quality of cardiac arrest process (e.g., increased use of defibrillation and ECPR), and better post-resuscitation process (e.g., targeted temperature management and early percutaneous coronary intervention). These processes have been emphasized in the American Heart Association Guidelines and European Resuscitation Council Guidelines for cardiopulmonary resuscitation in IHCA and OHCA [30, 31]. Because these guidelines are based on a large body of evidence for OHCA, future studies are needed to establish the evidence for IHCA.

Our study has some limitations. First, our definition of IHCA depended on administrative data for chest compression. A previous validation study on the database reported that most of the recorded procedures had sensitivity and specificity greater than 90%, but did not validate the records for chest compression [14]. Because we tried to exclude OHCA patients with cardiac arrest at admission using diagnosis codes with moderate sensitivity [14], some patients with OHCA may have been included in the study. Therefore, our estimates for the incidence of IHCA may have been biased. Because the sensitivity and specificity of the definition are unlikely to have changed over time, the observed trends are probably accurate. Second, we were not able to report the core variables for the cardiac arrest process of witnessed events, monitored cardiac arrest, event location, initial rhythm, and return of spontaneous circulation [15, 16]. We were also unable to obtain data regarding rapid-response systems, medical emergency teams, or “code blue” systems [32]. Therefore, we were unable to determine whether the cases of IHCA were preventable or non-preventable. Third, we were unable to report the long-term survival outcomes. We were also unable to obtain ideal neurological outcomes such as the Cerebral Performance Category [33]. Although we used the Japan Coma Scale at discharge instead [17], the association between Japan Coma Scale score at discharge and Cerebral Performance Category or modified Rankin Scale has not been determined in previous studies. As described above, this study of IHCA using data from an administrative inpatient database has many limitations. Considering the substantial burden and scant data regarding IHCA, there is a need to establish a nationwide Utstein-style prospective registry for IHCA in Japan. This would provide a structured framework for comparing systems of care, driving quality improvement, and facilitating identification of knowledge gaps and clinical research on IHCA [15, 16].

## Conclusion

In the present study, we found substantial associations between mortality and loss of health and IHCA in Japan. The incidence of IHCA was decreasing and the survival rate of IHCA was increasing over time.

## Supplementary Information

**Additional file 1: Table S1.** Number of patients at each fiscal year for overall and subgroup patients.

## Data Availability

The dataset analysed in the current study is not publicly available because of contracts with the hospitals providing data to the database.

## Abbreviations

IHCA: In-hospital cardiac arrest
OHCA: Out-of-hospital cardiac arrest
ICD-10: International Classification of Diseases, Tenth Revision
ECPR: Extracorporeal cardiopulmonary resuscitation

## References

[1] Andersen LW, Holmberg MJ, Berg KM, Donnino MW, Granfeldt A (2019). In-hospital cardiac arrest: a review. JAMA.

[2] Tunstall-Pedoe H, Bailey L, Chamberlain DA, Marsden AK, Ward ME, Zideman DA (1992). Survey of 3765 cardiopulmonary resuscitations in British hospitals (the BRESUS Study): methods and overall results. BMJ.

[3] Peberdy MA, Kaye W, Ornato JP, Larkin GL, Nadkarni V, Mancini ME (2003). Cardiopulmonary resuscitation of adults in the hospital: a report of 14720 cardiac arrests from the National Registry of Cardiopulmonary Resuscitation. Resuscitation.

[4] Iwami T, Nichol G, Hiraide A, Hayashi Y, Nishiuchi T, Kajino K (2009). Continuous improvements in ‘chain of survival’ increased survival after out-of-hospital cardiac arrests: a large-scale population-based study. Circulation.

[5] Kitamura T, Iwami T, Kawamura T, Nagao K, Tanaka H, Hiraide A, Implementation Working Group for the All-Japan Utstein Registry of the Fire and Disaster Management Agency (2010). Nationwide public-access defibrillation in Japan. N Engl J Med.

[6] Kitamura T, Kiyohara K, Sakai T, Matsuyama T, Hatakeyama T, Shimamoto T (2016). Public-access defibrillation and out-of-hospital cardiac arrest in Japan. N Engl J Med.

[7] Merchant RM, Yang L, Becker LB, Berg RA, Nadkarni V, Nichol G, American Heart Association Get with the Guidelines-Resuscitation Investigators (2011). Incidence of treated cardiac arrest in hospitalized patients in the United States. Crit Care Med.

[8] Nolan JP, Soar J, Smith GB, Gwinnutt C, Parrott F, Power S, National Cardiac Arrest Audit (2014). Incidence and outcome of in-hospital cardiac arrest in the United Kingdom National Cardiac Arrest Audit. Resuscitation.

[9] Sandroni C, Nolan J, Cavallaro F, Antonelli M (2007). In-hospital cardiac arrest: incidence, prognosis and possible measures to improve survival. Intensive Care Med.

[10] Morrison LJ, Neumar RW, Zimmerman JL, Link MS, Newby LK, McMullan PW, American Heart Association Emergency Cardiovascular Care Committee, Council on Cardiopulmonary, Critical Care, Perioperative and Resuscitation, Council on Cardiovascular and Stroke Nursing, Council on Clinical Cardiology, and Council on Peripheral Vascular Disease (2013). Strategies for improving survival after in-hospital cardiac arrest in the United States: 2013 consensus recommendations: a consensus statement from the American Heart Association. Circulation.

[11] Yokoyama H, Yonemoto N, Yonezawa K, Fuse J, Shimizu N, Hayashi T, J-RCPR Investigators (2011). Report from the Japanese registry of CPR for in-hospital cardiac arrest (J-RCPR). Circ J.

[12] Fujiwara S, Koike T, Moriyasu M, Nakagawa M, Atagi K, Lefor AK, IHCA Study Group (2016). A retrospective study of in-hospital cardiac arrest. Acute Med Surg.

[13] Yasunaga H (2019). Real world data in Japan: Chapter II the diagnosis procedure combination database. Ann Clin Epidemiol.

[14] Yamana H, Moriwaki M, Horiguchi H, Kodan M, Fushimi K, Yasunaga H (2017). Validity of diagnoses, procedures, and laboratory data in Japanese administrative data. J Epidemiol.

[15] Nolan JP, Berg RA, Andersen LW, Bhanji F, Chan PS, Donnino MW (2019). Cardiac arrest and cardiopulmonary resuscitation outcome reports: update of the utstein resuscitation registry template for in-hospital cardiac arrest: a consensus report from a task force of the International Liaison Committee on Resuscitation (American Heart Association, European Resuscitation Council, Australian and New Zealand Council on Resuscitation, Heart and Stroke Foundation of Canada, InterAmerican Heart Foundation, Resuscitation Council of Southern Africa, Resuscitation Council of Asia). Circulation.

[16] Nolan JP, Berg RA, Andersen LW, Bhanji F, Chan PS, Donnino MW, Utstein Collaborators (2019). Cardiac arrest and cardiopulmonary resuscitation outcome reports: update of the Utstein Resuscitation Registry Template for in-hospital cardiac arrest: a consensus report from a Task Force of the International Liaison Committee on Resuscitation (American Heart Association, European Resuscitation Council, Australian and New Zealand Council on Resuscitation, Heart and Stroke Foundation of Canada, InterAmerican Heart Foundation, Resuscitation Council of Southern Africa, Resuscitation Council of Asia). Resuscitation.

[17] Shigematsu K, Nakano H, Watanabe Y (2013). The eye response test alone is sufficient to predict stroke outcome—reintroduction of Japan Coma Scale: a cohort study. BMJ Open.

[18] Armitage P (1955). Tests for linear trends in proportions and frequencies. Biometrics.

[19] Jonckheere AR (1954). A distribution-free k-sample test against ordered alternatives. Biometrika.

[20] The Ministry of Health, Labour and Welfare. Japan statistical surveys 2017. The Ministry of Health, Labour and Welfare, Japan; 2017. https://www.mhlw.go.jp/english.html.

[21] Fire and Disaster Management Agency. Report on a study on social system development to improve survival from emergency cardiovascular disease. https://www.fdma.go.jp/publication/rescue/post7.html. **(in Japanese)**

[22] Holmberg MJ, Ross CE, Fitzmaurice GM, Chan PS, Duval-Arnould J, Grossestreuer AV, American Heart Association’s Get With The Guidelines–Resuscitation Investigators (2019). Annual incidence of adult and pediatric in-hospital cardiac arrest in the United States. Circ Cardiovasc Qual Outcomes.

[23] WHO Mortality Database—WHO. https://www.who.int/data/data-collection-tools/who-mortality-database.html.

[24] Ministry of Health, Labour and Welfare. https://www.mhlw.go.jp/english/database/db-hw/vs01.html.

[25] Makino J, Fujitani S, Twohig B, Krasnica S, Oropello J (2014). End-of-life considerations in the ICU in Japan: ethical and legal perspectives. J Intensive Care.

[26] Jones DA, DeVita MA, Bellomo R (2011). Rapid-response teams. N Engl J Med.

[27] Maharaj R, Raffaele I, Wendon J (2015). Rapid response systems: a systematic review and meta-analysis. Crit Care.

[28] Girotra S, Nallamothu BK, Spertus JA, Li Y, Krumholz HM, Chan PS, American Heart Association Get with the Guidelines–Resuscitation Investigators (2012). Trends in survival after in-hospital cardiac arrest. N Engl J Med.

[29] Schluep M, Gravesteijn BY, Stolker RJ, Endeman H, Hoeks SE (2018). One-year survival after in-hospital cardiac arrest: a systematic review and meta-analysis. Resuscitation.

[30] Merchant RM, Topjian AA, Panchal AR, Cheng A, Aziz K, Berg KM, Adult Basic and Advanced Life Support, Pediatric Basic and Advanced Life Support, Neonatal Life Support, Resuscitation Education Science, and Systems of Care Writing Groups (2020). Part 1: executive summary: 2020 American Heart Association Guidelines for Cardiopulmonary Resuscitation and Emergency Cardiovascular Care. Circulation.

[31] Perkins GD, Graesner JT, Semeraro F, Olasveengen T, Soar J, Lott C, European Resuscitation Council Guideline Collaborators (2021). European Resuscitation Council guidelines 2021: executive summary. Resuscitation.

[32] Sebat F, Musthafa AA, Johnson D, Kramer AA, Shoffner D, Eliason M (2007). Effect of a rapid response system for patients in shock on time to treatment and mortality during 5 years. Crit Care Med.

[33] Jennett B, Bond M (1975). Assessment of outcome after severe brain damage. Lancet.

