# Supplementary material for: Incidence and outcomes of in-hospital cardiac arrest in Japan 2011–2017: a nationwide inpatient database study
Source: J Intensive Care. 2022 Mar 3;10:10. doi: 10.1186/s40560-022-00601-y (PMC8895772; doi:10.1186/s40560-022-00601-y)
Supplement: Supplementary file 1 — Additional file 1: Table S1. Number of patients at each fiscal year for overall and subgroup patients. [file 40560_2022_601_MOESM1_ESM.docx]

*Additional file 1*

**Incidence and outcomes of in-hospital cardiac arrest in Japan 2011–2017: a nationwide inpatient database study**

Hiroyuki Ohbe, Takashi Tagami, Kazuaki Uda, Hiroki Matsui, Hideo Yasunaga

**Table S1** Number of patients at each fiscal year for overall and subgroup patients

|  |  | Fiscal year | | | | | | |
| --- | --- | --- | --- | --- | --- | --- | --- | --- |
|  | Total | 2011 | 2012 | 2013 | 2014 | 2015 | 2016 | 2017 |
| Overall | 274664 | 41263 | 39810 | 35927 | 39866 | 38734 | 40827 | 38237 |
| Patients with defibrillation | 45675 | 6951 | 6653 | 5954 | 6642 | 6447 | 6691 | 6337 |
| Patients without defibrillation | 228989 | 34312 | 33157 | 29973 | 33224 | 32287 | 34136 | 31900 |
| Patients with ECPR | 9203 | 1011 | 1137 | 1184 | 1354 | 1458 | 1500 | 1559 |
| Age category |  |  |  |  |  |  |  |  |
| Aged <1 year | 3077 | 398 | 475 | 499 | 477 | 427 | 439 | 362 |
| Aged 1–18 years | 2661 | 336 | 408 | 376 | 407 | 406 | 396 | 332 |
| Aged 19–64 years | 52547 | 8310 | 8126 | 7233 | 7541 | 7184 | 7346 | 6807 |
| Aged 65–74 years | 58197 | 8290 | 8264 | 7680 | 8555 | 8569 | 8651 | 8188 |
| Aged 75–84 years | 90190 | 14122 | 13361 | 11866 | 12975 | 12566 | 13163 | 12137 |
| Aged >84 years | 67992 | 9807 | 9176 | 8273 | 9911 | 9582 | 10832 | 10411 |
| Illness category |  |  |  |  |  |  |  |  |
| Medical-cardiac | 80657 | 11130 | 11004 | 10283 | 11630 | 11548 | 12669 | 12393 |
| Medical-noncardiac | 151051 | 24204 | 22452 | 19869 | 21948 | 21004 | 21781 | 19793 |
| Surgical-cardiac | 7797 | 977 | 1135 | 1042 | 1131 | 1184 | 1156 | 1172 |
| Surgical-noncardiac | 17199 | 2592 | 2668 | 2387 | 2455 | 2416 | 2389 | 2292 |
| Obstetric | 144 | 18 | 21 | 25 | 20 | 20 | 22 | 18 |
| Trauma | 17816 | 2342 | 2530 | 2321 | 2682 | 2562 | 2810 | 2569 |

Each fiscal year started on 01 April and ended on 31 March.

ECPR, extracorporeal cardiopulmonary resuscitation.
